# Enhancement of Photodetective Properties on Multilayered MoS_2_ Thin Film Transistors via Self-Assembled Poly-L-Lysine Treatment and Their Potential Application in Optical Sensors

**DOI:** 10.3390/nano11061586

**Published:** 2021-06-17

**Authors:** Seung Gi Seo, Jae Hyeon Ryu, Seung Yeob Kim, Jinheon Jeong, Sung Hun Jin

**Affiliations:** Department of Electronic Engineering, Incheon National University, Incheon 22012, Korea; imyohanzzang@gmail.com (S.G.S.); kecjoel@gmail.com (J.H.R.); scape11@naver.com (S.Y.K.); suytwer3420@naver.com (J.J.)

**Keywords:** a-Si:H TFT, MoS_2_ TFT, photodetector, comparative study, molecular doping, poly-L-lysine

## Abstract

Photodetectors and display backplane transistors based on molybdenum disulfide (MoS_2_) have been regarded as promising topics. However, most studies have focused on the improvement in the performances of the MoS_2_ photodetector itself or emerging applications. In this study, to suggest a better insight into the photodetector performances of MoS_2_ thin film transistors (TFTs), as photosensors for possible integrated system, we performed a comparative study on the photoresponse of MoS_2_ and hydrogenated amorphous silicon (a-Si:H) TFTs. As a result, in the various wavelengths and optical power ranges, MoS_2_ TFTs exhibit 2~4 orders larger photo responsivities and detectivities. The overall quantitative comparison of photoresponse in single device and inverters confirms a much better performance by the MoS_2_ photodetectors. Furthermore, as a strategy to improve the field effect mobility and photoresponse of the MoS_2_ TFTs, molecular doping via poly-L-lysine (PLL) treatment was applied to the MoS_2_ TFTs. Transfer and output characteristics of the MoS_2_ TFTs clearly show improved photocurrent generation under a wide range of illuminations (740~365 nm). These results provide useful insights for considering MoS_2_ as a next-generation photodetector in flat panel displays and makes it more attractive due to the fact of its potential as a high-performance photodetector enabled by a novel doping technique.

## 1. Introduction

For achieving the ultimate scaling of Si devices below a sub-nanometer, direct tunneling of leakage current in the channel is a critical problem to be resolved, because it can lead to large power consumption [1,2]. In this regard, transition metal dichalcogenides (TMDCs) with a sizable and tunable energy bandgap, depending on the number of layers, have actively been studied as a potential candidate for the next generation of semiconductors. In addition, the versatile properties of TMDCs, including outstanding electrostatic gate coupling, no dangling bonds, high effective mass of electrons leading to less direct tunneling leakage, and unique optical/chemical properties, have generated plenty of research interest [3,4,5,6]. Among the varieties of the TMDC family, molybdenum disulfide (MoS_2_) is a representative of TMDC material with easy preparation via CVD and/or exfoliation, high electron mobility (>100 cm^2^/Vs), and an abundant form of MoS_2_ as natural minerals [7,8]. Until now, enormous research activities, involving basic transport mechanisms, circuit level demonstration for flat panel displays and FinFETs, new concepts of devices, and gas sensors, have been extensively performed [9,10,11,12,13,14,15,16,17]. In particular, among the numerous research fields of MoS_2_ thin film transistors (TFTs), applications for photodetectors and display backplane transistors have been regarded as important and promising topics for the future technology with its novel electrical/optical properties [18,19,20]. Those studies for MoS_2_ photodetectors have reported remarkable performances including broad band detection from ultraviolet to near-infrared (NIR), ultra-high photoresponsivity, flexible application, polarization sensitive photodetection, high-speed photoresponse, high spatially resolved imaging [21,22,23,24,25,26,27]. However, most of the studies have focused on the performances of the MoS_2_ photodetector itself or emerging applications using them because of the easy preparation of the devices via exfoliated or CVD layers, their relatively stable device performance, and reproducible device implementation as compared with other TMDC layers. In order to suggest a better insight into photodetective properties for MoS_2_ thin film transistors (TFTs) quantitatively as photosensors, possibly integrated with a display system, in this study, we carried out a comparative study on the photoresponse of the MoS_2_ TFTs and hydrogenated amorphous silicon (a-Si:H) TFTs. This was due to the immediate availability of highly accumulated information in the literature in terms of the optical properties of a-Si:H-based TFTs and their understanding on device behaviors. a-Si:H has been used as conventional TFTs in the display industry, and photodetectors integrated with active matrix are conjugated as imaging scanners, optical feedback systems, or/and touch panels for interactive display operation [28,29,30,31]. Compared to a-Si:H TFTs, MoS_2_ TFTs have demonstrated potential for both display backplane TFTs and photodetectors [18,19,20,21,22,23,24,25,26,27]. Furthermore, even with comparison of amorphous indium–gallium–zinc-oxide (a-IGZO) TFTs, which are presently one of the most promising display backplane TFTs with high mobility and maturity of material, MoS_2_ TFTs possess advantages of detecting spectrum and negligible photo persistent current (PPC) effects [31,32]. Thus, this comparative study was expected to provide intuitive criteria for researchers in terms of the key aspects that are required for MoS_2_ layers as one of the next-generation semiconductors for the future photodetectors in display systems.

Furthermore, as a strategy to improve the field effect mobility and photoresponse of the MoS_2_ TFTs, we adopted a doping treatment in this work. Until now, various doping methods have been applied including high-k-based fixed-charge induction, molecule-induced charge transfer, organic solvents-based doping, electrical doping via local electrode configuration, electrothermal doping, and gas-based doping. Among these techniques, molecular doping has been predominantly utilized owing to the immediately accessible approach with several advantages such as post process compatibility, unnecessity to deposit additional layers, and low temperature during the treatment on devices. In this regard, molecular doping by poly-L-lysine (PLL) treatment was applied to the MoS_2_ TFTs. In general, PLL with amino group has been frequently used to make graphene and carbon nanotubes positively functionalized, which results in high affinity to induce negatively charged biomolecules [33,34,35]. This technique leads to the improvement on uniformity of nanomaterials coating and its corresponding control of sequence order associated with electrical polarity of molecule ending groups. On the other hand, PLL-based doping for TMDC and its corresponding evaluation on optical detectivity control, compared with conventional TFTs, for example, a-Si:H TFTs, has been reported to be limited. In this study, PLL was coated on the surface of MoS_2_ layers to enhance the electron charges in the MoS_2_ TFTs. Transfer and output characteristics of the MoS_2_ TFTs clearly showed improved photocurrent generation under a wide range of illumination (740~365 nm). In addition, its chemical and physical properties were analyzed by X-ray spectroscopy (XPS), Raman spectroscopy, and atomic force microscopy (AFM) to confirm the doping’s effects on the MoS_2_ TFTs. As a result, along with the extracted photo responsivity and detectivity, PLL treatment demonstrated not only that it induced n-doping effects but also that it improved the photoresponse of the MoS_2_ TFTs.

These results will provide useful insights for considering MoS_2_ as a next-generation photodetector in flat panel displays and make it more attractive as a potential high-performance photodetector enabled by a novel doping technique.

## 2. Materials and Methods

**Figure 1:**
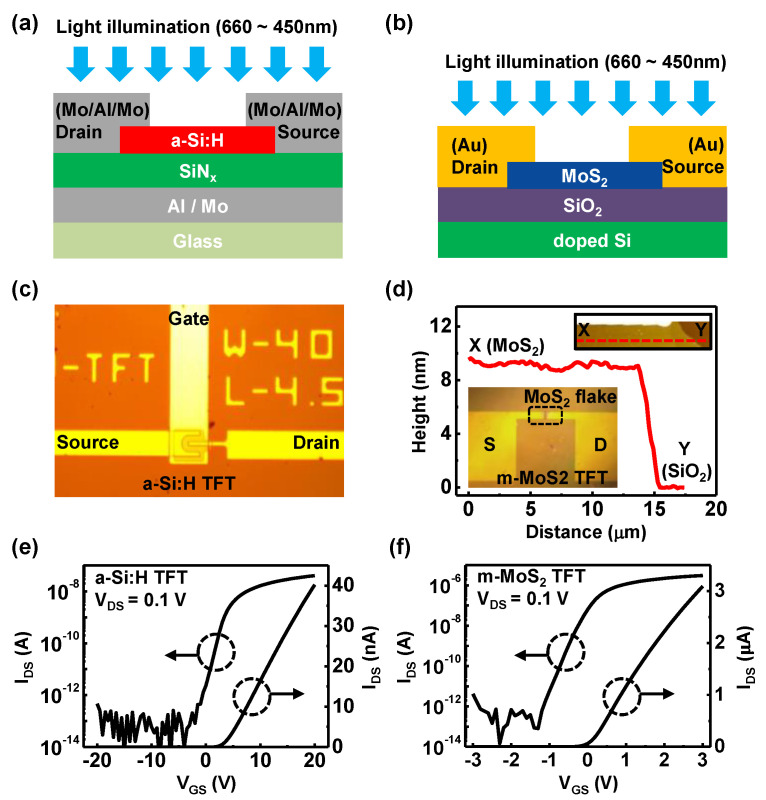
Cross-sectional views of (**a**) an a-Si:H TFT and (**b**) a m-MoS_2_ TFT under illumination. Optical microscope images of (**c**) an implemented a-Si:H TFT. (**d**) Topographical cross-sectional profile along the dashed line indicates in the atomic force a microscope image of exfoliated MoS_2_ layers, X on thermal oxide and Y in the FETs. X and Y in the inset (right top) denote the location of an oxide (SiO_2_) and a MoS_2_ layer, respectively. Inset (left bottom) shows optical microscope images of the implemented m-MoS_2_ TFT. The channel width-to-length ratio (W/L) was 40/4.5 μm for the a-Si:H TFT and 30/10 μm for the m-MoS_2_ TFT. Transfer characteristics of (**e**) a-Si:H TFT and (**f**) m-MoS_2_ TFT in linear and log scale measured at V_DS_ = 0.1 V.

Two kinds of photosensitive TFTs were fabricated as shown in Figure 1: (i) multilayered MoS_2_ (m-MoS_2_) TFT and (ii) a-Si:H TFT. Figure 1a shows an inverted staggered a-Si:H TFT composed of a 360 nm thick Al/Mo gate metal on a glass substrate, a 430 nm thick SiN_x_ gate insulator, a 210 nm thick a-Si:H/n+a-Si:H layer, and a 430 nm thick Mo/Al/Mo source and drain contacts, which were based on one of the conventional process protocols for the implementation of a-Si:H fabrication in the TFT-LCD mass production lines. Figure 1b shows m-MoS_2_ TFTs that were implemented on the thermally oxidized Si wafers with 10 nm thick SiO_2_. The heavily phosphorus-doped silicon wafer (*ρ* ~ 0.005 ohm·cm) was initially used as a global gate. The multilayers of MoS_2_ were mechanically exfoliated from bulk MoS_2_ crystals (SPI Supplies, 429ML-AB, West Chester, PA, USA) and transferred onto Si substrates, with thermal oxide (~10 nm) as a gate insulator, using adhesive poly dimethylsiloxane (PDMS) elastomer. Then, immediate annealing was performed in mixed gas (~Ar/H_2_) at a temperature of 400 °C for 1 h to remove organic residues and surface treatment on the MoS_2_ films that might contaminate them during the transfer process [36]. Thereafter, 35 nm Au was evaporated using e-gun evaporators, followed by lifting off on a photo lithographically patterned area, forming the source/drain electrodes. Figure 1c displays an optical microscope image of fabricated a-Si:H TFT with a 4.6 μm channel length and a 40 μm channel width. Figure 1d exhibits the height profile of the m-MoS_2_ by atomic force microscopy (AFM, Bruker, MULTIMODE-8-AM, Billerica, MA, USA), confirmed as ~9.5 nm (corresponding to ~15 layers). The insets display optical microscope images of fabricated m-MoS_2_ TFT with a 10 μm channel length and a 30 μm channel width.

For n-type doping, poly-L-lysine (PLL) was purchased from Sigma–Aldrich (Seoul, Korea) and used as charge enhancing molecules for the m-MoS_2_ TFTs. For the treatment, the fabricated m-MoS_2_ TFTs were dipped in a water solution containing PLL (0.1% *w*/*v*) in ambient air, at room temperature. The dipping time was 2.5 h for the PLL treatment, followed by baking at 100 °C on a hot plate for 10 min. A Raman spectrometer (WITEC alpha300, Ulm, Germany) was used to analyze the Raman spectra and PL intensity of the TMDC flakes with a 532 nm laser excitation and 1 μm of beam size. Light for comparison of the photo response between a-Si:H and m-MoS_2_ TFTs was illuminated by the laser (wavelength: 630 nm, 530 nm, and 450 nm; power: 1 μW~20 mW) and a multi-wavelength fiber-coupled LED source (Mightex, Inc., Pleasanton, CA, USA) with various wavelength (365 nm, 455 nm, 530 nm, 656 nm, and 740 nm), and 3 mW cm^−2^ of power density was employed to measure the photocurrent of m-MoS_2_ TFTs before and after PLL treatment. All the electrical characterizations were measured with a semiconductor impedance analyzer (Agilent 4155C, Seoul, Korea) in ambient air at room temperature.

## 3. Results and Discussion

### 3.1. Comparative Study on the Photoresponse of a-Si:H and m-MoS_2_ TFTs

Figure 1e,f display the transfer characteristics (I_DS_–V_GS_) of implemented a-Si:H TFTs and m-MoS_2_ TFTs at V_DS_ = 0.1 V. The on/off current ratio was ~10^6^ for the a-Si:H TFT and ~10^7^ for the m-MoS_2_ TFT. The field-effect mobility (μ_FE_) was, respectively, extracted as 0.34 and 12.36 cm^2^/V·s for a-Si:H and m-MoS_2_ TFTs from the equation μ_FE_ = g_m_·L/(C_OX_·V_DS_·W), where g_m_ is the transconductance, C_OX_ is the dielectric capacitance, L and W indicate channel length and width, and V_DS_·is drain-to-source voltage, respectively. The subthreshold swing (SS) of the m-MoS_2_ transistor was ~200 mV/dec, while it was 1 V/dec with the a-Si:H TFTs. The extracted electrical properties indicate that the quality of fabricated TFTs was reasonable compared with previous results in the literature [31,37,38,39]; thus, the photoresponse of both TFTs were ready to be compared without consideration of noticeable defects or flaws.

**Figure 2:**
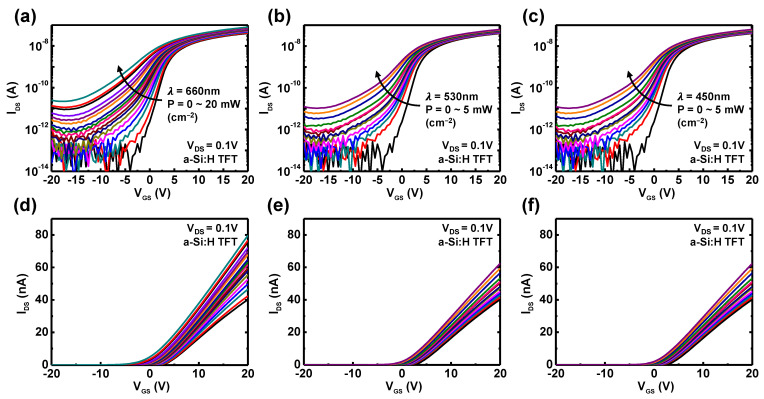
Transfer characteristics of a-Si:H TFTs under illumination of 660, 530, and 450 nm lasers in (**a**–**c**) log and (**d**–**f**) linear scale. Various optical intensities (0~5 mW cm^–2^) were applied to a-Si:H TFTs with the same distance and angle.

**Figure 3:**
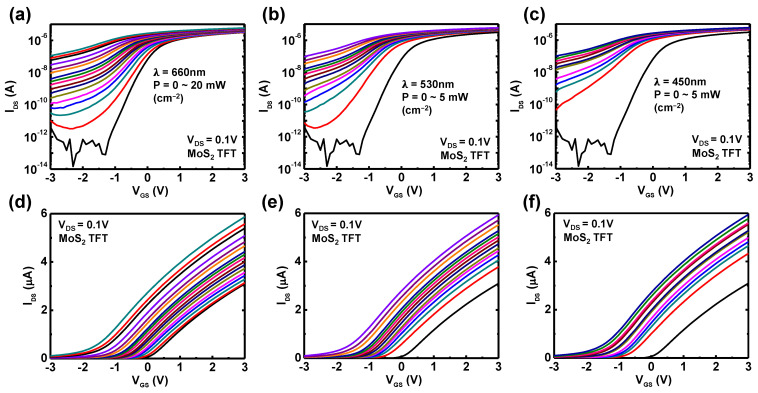
Transfer characteristics of m-MoS_2_ TFTs under illumination at 660, 530, and 450 nm using lasers in (**a**–**c**) log and (**d**–**f**) linear scale at V_DS_ = 0.1 V. Various optical intensities (0~5 mW cm^–2^) were applied to a-Si:H TFTs with the same distance and angle.

For a fair comparison, both TFTs had similar channel dimensions, the distance between light source and device was fixed, and the duration of illuminated light was identically set. Then, to investigate the photoelectrical properties of the a-Si:H and m-MoS_2_ TFTs, the transfer characteristics under illumination with different wavelength and intensities were measured. Figure 2a–c show photo-induced transfer curves at V_DS_ = 0.1 V, obtained from a-Si:H TFT in log scale, and their linear scale is shown in Figure 2d–f. Power intensity varies from 1 μW to 20 mW for 660 nm laser, from 1 μW to 5 mW for 530 nm laser, and from 10 μW to 5 mW for 450 nm laser. Measured data, as shown in Figure 2, revealed the generation of photocurrent (I_photo_ = I_light_ − I_dark_) of a-Si:H TFTs under illumination, corresponding to all wavelengths in this test. The generation of a photocurrent can be explained according to the following scenario: when the light illuminated the channel materials, photon energy of light produced the electron–hole pairs in the channel region. Then, the excited electron–hole pairs drifted along the channel by the applied lateral E-field associated with V_DS_, resulting in the increase of drain-to-source current. In addition, the photocurrent of a-Si:H was enhanced as the light intensity increased, that is, a stronger optical power generates more electron–hole pairs from the channel materials. Figure 2 presents a proper photo-transistor operation of a-Si:H TFTs, and the linear scale graphs indicate a gradual photocurrent increase at the on-state (V_GS_ > 0 V) without degradation of the field-effect mobility. With the same procedures for the evaluation of the photoresponse, Figure 3 shows the transfer characteristics of the m-MoS_2_ TFTs under illumination with different wavelength and intensities. The m-MoS_2_ TFTs also exhibit proper photo-transistor operation, and a parallel shift of V_th_ was observed in the linear scale graphs. When we visually compared the photoresponse for both TFTs, it can be directly seen that the photocurrent of the a-Si:H TFTs was at an nA level, whereas the m-MoS_2_ TFTs was at a μA level. Furthermore, the m-MoS_2_ TFTs had a sensitive dependency on the wavelength of light, and these photoresponses can be potentially tailored by engineering the layer thickness [40,41]. Thus, these results elucidate a better potential of MoS_2_ for the versatile and high-performance photodetectors.

**Figure 4:**
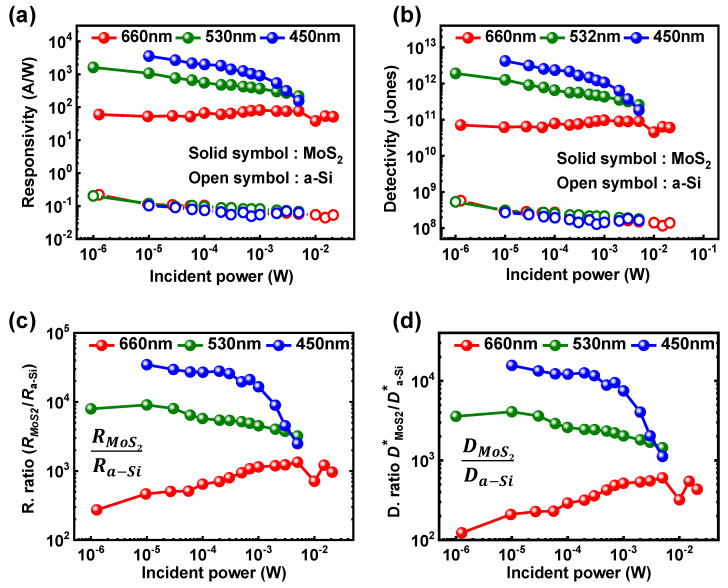
(**a**) Responsivities and (**b**) detectivities of a-Si:H and m-MoS_2_ TFTs extracted from Figure 2 and Figure 3. Directly compared (**c**) responsivities and (**d**) detectivities of a-Si:H and m-MoS_2_ TFTs. The ratio was calculated from the values of the m-MoS_2_ TFTs divided by those of the a-Si:H TFTs.

One of the most important figures of merit for a photodetector is its external photoresponsivity and detectivity. To quantitatively compare the performance of photoresponse, both photoresponsivity and detectivity were extracted and shown in Figure 4. The photoresponsivity and detectivity were calculated from equations R = I_photo_/P_light_, D = R × S^1/2^(2qI_dark_)^1/2^, where P is a total incident optical power, S is the effective illuminated area, q is the electron charge, and I_dark_ is dark current. I_photo_ and I_dark_ are the current levels in the off regime, for the bias condition, at V_GS_ = −1.2 V for m-MoS_2_ TFTs and V_GS_ = −1.5 V for a-Si:H TFTs, respectively, under either light illumination or dark condition. Figure 4a shows extracted photoresponsivity of m-MoS_2_ and a-Si:H TFTs. The photoresponsivities of the m-MoS_2_ TFTs ranged from 10^2^ to 10^4^ for RGB light, and those of the a-Si:H TFTs were within 10^−1^, wherein the responsivities of the a-Si:H TFTs were similar regardless of the wavelength as reported in the literature [31]. In the all of wavelengths and optical power ranges, the m-MoS_2_ TFTs exhibited 2~5 orders larger responsivities than that of the a-Si:H TFTS, which is attributed to different photocurrent value for both TFTs. In addition, Figure 4b presents the detectivity of the m-MoS_2_ and a-Si:H TFTs, from which detectivities of the m-MoS_2_ TFTs were 10^11^~10^13^ and those of the a-Si:H TFTs were extracted as 10^8^~10^9^. Due to the noticeable current ratio between I_photo_ and I_dark_, the m-MoS_2_ TFTs had clearly higher detectivities than a-Si:H TFTs. As a result, in the all of wavelength and optical power range, the m-MoS_2_ TFTs exhibited 2~4 orders larger detectivities. The overall quantitative comparison of responsivities and detectivities for the m-MoS_2_ and a-Si:H TFTs are summarized in Figure 4c,d, which obviously confirms a much better performance of MoS_2_ photodetector.

**Figure 5:**
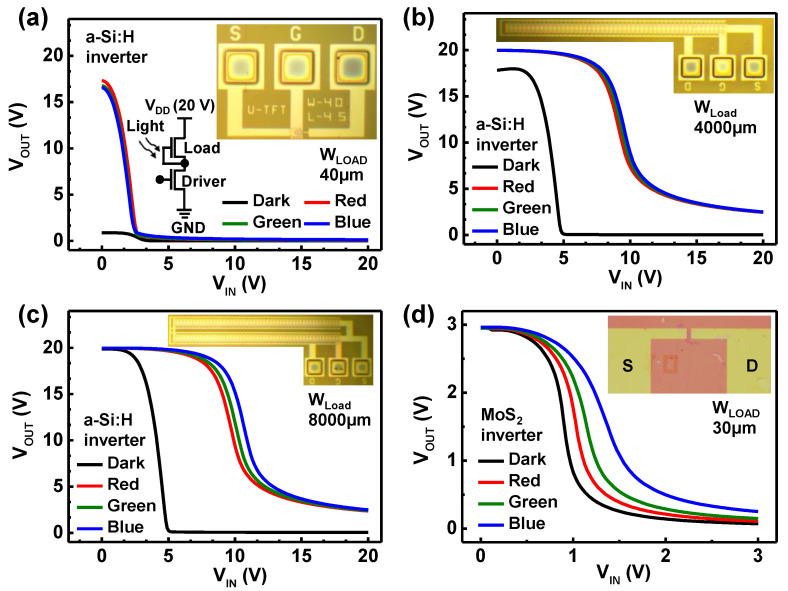
Voltage transfer characteristics of a-Si:H DLED inverters with (**a**) 40 μm, (**b**) 4000 μm, and (**c**) 8000 μm of W_load_ under illumination from dark to blue. (**d**) Voltage transfer characteristics of m-MoS_2_ DLED inverters with 30 μm W_load_. Insets show optical image of a-Si:H and m-MoS_2_ TFTs with each channel dimension.

In addition, as a circuit level photodetector, photosensitive inverters were implemented and compared using a-Si:H and m-MoS_2_ TFTs in Figure 5. These photoinverters are key component of light-to-frequency conversion circuits (LFCs), which are practically beneficial for the future IoT systems required for a high level of security [37,38,39,42]. To demonstrate the capabilities of a-Si:H and m-MoS_2_ TFTs in a circuit-level photodetector, the photo response of depletion load enhancement driver (DLED) inverters was measured. In the configuration of an a-Si:H DLED inverter, the channel width of the load TFT (W_load_) was varied to change the effective illuminated area of the a-Si:H channel under light illumination. Figure 5a displays the voltage transfer characteristics (VTCs) of an a-Si:H DLED inverter with 40 μm of W_load_. In dark conditions, the switching behavior of the inverter was poor, whereas it is improved under light illumination. This is because sufficient load currents are required to obtain reasonable swing performance in VTCs for a DLED inverter. Thus, under illumination, increased currents yielded improved switching behaviors. However, in this case, VTC curves were not distinguished depending on different wavelength of light (i.e., R, G, B), so that the requirements of photosensitive inverters were not satisfied. To secure sufficient load currents, a-Si:H DLED inverters with large W_load_ of 4000 and 8000 μm were utilized. As shown in Figure 5b,c, in dark conditions, decent switching properties were obtained with 4000 μm W_load_ and full swing characteristics were achieved with 8000 μm W_load_. Furthermore, minimum V_OUT_ (V_OL_) values did not reach the value of zero due to the fact of too much photocurrent stemming from a large illuminating area; thus, there waw a tradeoff between the stability of the inverter operation and photoresponse. On the contrary, the DLED inverter composed of m-MoS_2_ TFTs with 30 μm W_load_ exhibited stable full-swing characteristics under dark and illumination at all wavelengths, which is attributed to better mobility and photoresponse than the a-Si:H TFTs. Consequently, Figure 5 indicates that the a-Si:H TFTs require at least 8000 μm of channel width for the application of photosensitive inverters, compared to the 30 μm of the m-MoS_2_ TFTs. These results elucidate that m-MoS_2_ TFTs have robust advantages with regard to the level of device integration per area, chip density, and sensitive modulation properties in variations of wavelength from dark to blue compared to the a-Si:H TFTs.

### 3.2. Improvement in the Photoresponse of m-MoS_2_ TFTs by Molecular Doping Technique

In addition to revealing the high optoelectrical performance of m-MoS_2_ TFTs by comparison with a-Si:H TFTs, the photoresponsivity can be enhanced by molecular doping treatment [43,44,45]. Although there are different techniques to improve the photodetector’s performance, the molecular doping method has several advantages, as it does not need a change in the device’s structure or the addition of different channel materials or layers but consist of a low temperature process, ultra-thin thickness, and post-process compatibility after device fabrication. In this regard, poly-L-lysine (PLL) was adopted to enhance the photoresponsivity via n-doping effect for m-MoS_2_ TFTs from PLL molecules.

**Figure 6:**
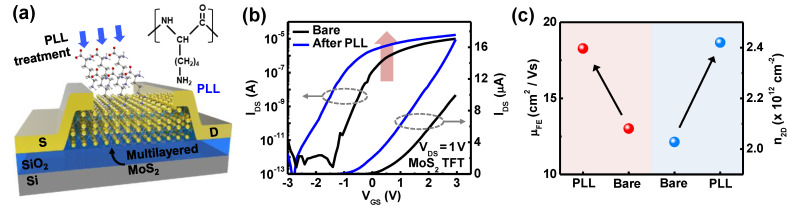
(**a**) A schematic illustration of m-MoS_2_ TFTs with PLL molecules treated on the surface of m-MoS_2_; (**b**) transfer characteristics of m-MoS_2_ TFTs at V_DS_ = 1 V before and after PLL treatment; (**c**) extracted field effect mobility and carrier concentration before and after PLL treatment.

Figure 6a illustrates a schematic for molecular doping of m-MoS_2_ TFT to induce donor-like doping with facilitation of charge enhancers. Attached on the surface of m-MoS_2_, amine (NH_2_)-based charge transfer of PLL molecules can play a role in donating electrons toward MoS_2_ layers, leading to donor-like doping effects with protonated NH_3_^+^ functional group in PLL. Figure 1b shows transfer characteristics of m-MoS_2_ TFTs at V_DS_ = 1 V in log and linear scale, respectively. After the PLL treatment for 1.5 h, without discernible degradation of SS and of-off ratio, clear V_th_ shift (ΔV_th_ ~ −1.0 V) and improvement of on-current were shown. For the quantitative analysis on doping effects according to the PLL treatment, mobility, and carrier concentration was plotted in Figure 6c. The carrier concentration was calculated by the following equation n_2D_ = (L/W) × (I_DS_/q·μ_FE_·V_DS_), where L and W are the length and width of channel and q is the electron charge. As a consequence of charge enhancement, μ_FE_ was extracted as 13.0 and 18.3 cm^2^/V·s before and after PLL treatment, respectively, which reveals a 40% increase in μ_FE_. In parallel, n_2D_ was calculated as 2.0 × 10^12^ and 2.4 × 10^12^ before and after PLL treatment, respectively, from which 20% enhancement of n_2D_ was obtained. These results clearly show the improvement of electrical properties by treatment of PLL.

**Figure 7:**
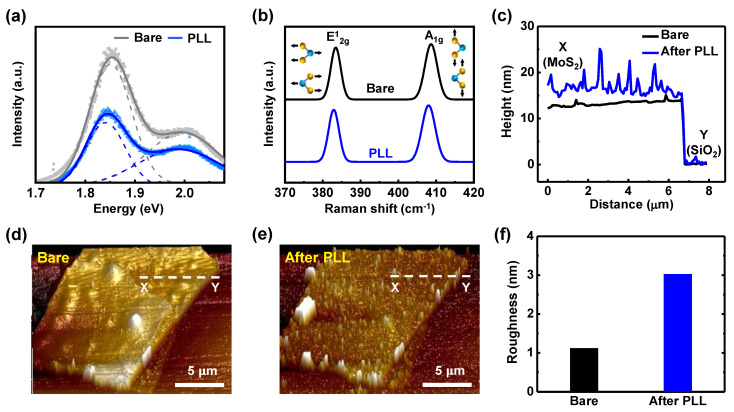
(**a**) Photoluminescence (PL) characteristics of m-MoS_2_ flakes before and after PLL treatment; (**b**) evolution of Raman spectra of m-MoS_2_ corresponding to bare condition and PLL treatment; (**c**) Height profile of m-MoS_2_ flakes measured by AFM before and after PLL treatment. X and Y denote MoS_2_ and SiO_2_ region, respectively; (**d**) AFM topographical images of m-MoS_2_ flake for (**d**) bare condition and (**e**) PLL treatment. Dashed line indicates extracted points to obtain height profile in Figure 7c; (**f**) evolution of surface roughness of the m-MoS_2_ flakes extracted from the AFM topography. The Surface roughness increased via attached molecules after PLL treatment.

Thereafter, for better understanding on electrically observed doping effects by PLL, chemical and physical properties and their analysis on m-MoS_2_ TFTs were examined. First, optical properties were examined via photoluminescence (PL) spectroscopy. Figure 7a shows PL spectra of m-MoS_2_ TFTs after PLL treatment, from which peak intensity after PLL treatment was reduced because donor-like doping effects enhanced the formation of tightly bound trions of MoS_2_ [45,46,47], leading to a decrease in the radiative recombination of excitons. With the extracted optical properties, electrical behaviors, corresponding to each optical excitation, were investigated in the following section. As reported elsewhere [45,47,48,49,50,51,52,53,54], Raman spectroscopy also has been dominantly used as a reliable tool to confirm the doping effects on TMDCs. Figure 4a plots the Raman spectra of the m-MoS_2_ TFTs before and after the treatment of PLL. In the Figure 4a, two characteristic vibrational modes (E^1^_2g_ and A_1g_) were observed near 383 and 409 cm^−1^ in the bare m-MoS_2_ flakes, where E^1^_2g_ mode was attributed to the in-plane vibration between Mo and S atoms, whereas the A_1g_ mode was due to the out-of-plane vibration between Mo and S atoms. After the PLL treatment, the E^1^_2g_ and A_1g_ peaks were shifted left by 0.25 cm^−1^ and 0.63 cm^−1^ for m-MoS_2_ TFTs, respectively. Obviously observed left shifts of two characteristics mode in Raman peaks describe that n-doping effects of PLL treatment result from the increase of the electron–phonon scattering due to the higher electron concentration [45,47,48,49,51,52,55]. Thus, PLL treatment would enhance the electron density, leading to increase of electron–phonon scattering, which lends phonon frequency decreased [54]. The Raman results support the n-doping effects on m-MoS_2_ TFTs, which are attributed to the PLL treatment, and their chemical analysis via Raman nicely matched with the entire trend of electrical properties as shown in Figure 6.

In addition, as for the physical examination, morphological change of m-MoS_2_ flakes by PLL treatment was measured by atomic force microscopy (AFM). Figure 7c–e display the height information and AFM 3D images of MoS_2_ flake before and after PLL treatment for 3 h. Figure 7c reveals the increased height profile of MoS_2_ flake along the flake-substrate line, which is attributed to attached PLL molecules on the surface of MoS_2_ flake. The position of X (MoS_2_) and Y (SiO_2_) measured by AFM are denoted in Figure 7d,e. Moreover, the images of Figure 7d,e show the bare and PLL-treated MoS_2_ flake, respectively, where the bumpy surface of MoS_2_ flakes was slightly observed after PLL treatment. Thereafter, as a root mean square (RMS) value, surface roughness by AFM analysis was extracted as 1.12 nm (or 3.03 nm) for MoS_2_ flakes before (or after) PLL treatment for 3 h, respectively (Figure 4f). All results in Figure 7c–f are one of reasonable evidence to validate that the attachment of PLL molecules enables to modify nano-scaled surface morphology of MoS_2_ flake [51,56], resulting in possible tuning of electrical properties for m-MoS_2_ FFTs.

**Figure 8:**
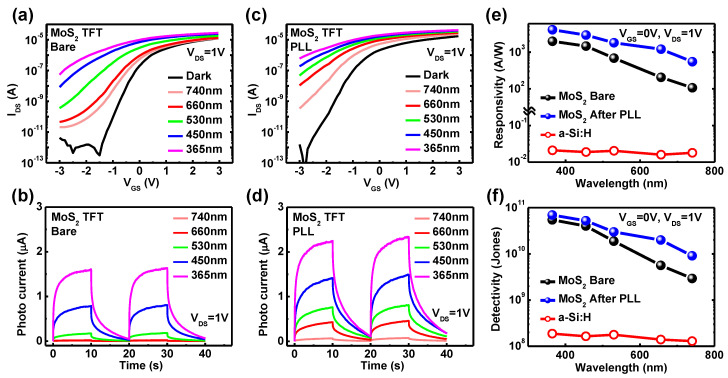
Photoresponses from (**a**) transfer characteristics and (**b**) two-terminal output characteristics of bare m-MoS_2_ TFTs under illumination from 740 nm to 365 nm. Photoresponses from (**c**) transfer characteristics and (**d**) two-terminal output characteristics of PLL-treated m-MoS_2_ TFTs. Comparisons of (**e**) responsivities and (**f**) detectivities of bare and PLL-treated m-MoS_2_ TFTs and a-Si:H TFTs extracted at V_GS_ = 0 V, V_DS_ = 1 V for wavelength from 740 nm to 365 nm.

To validate the doping effects on improvement of photoresponse, variations of currents under illumination were monitored before and after PLL treatment. Figure 8a presents transfer characteristics (e.g., 3-terminal operation) of the m-MoS_2_ TFTs without any doping treatment. Under illumination from the visible to UV range (740~365 nm), an increase in currents was observed in the bare m-MoS_2_ TFTs, which are commonly reported behaviors in the literature [37,38,39,40]. Furthermore, an increase in the current was gradually enhanced as the wavelength of the light decrease (i.e., as photon energy increase) [37,38,39,40]. Thereafter, time-dependent photoresponse in two-terminal operation was measured. Figure 8b shows photocurrents of bare m-MoS_2_ TFTs at V_DS_ = 1 V. As similar with transfer characteristics in Figure 8a, increases in the photocurrent are gradually shown as the wavelengths of the light decreased. Then, after PLL treatment for bare the m-MoS_2_ TFTs, the photoresponses were measured with same condition. Figure 8c displays the transfer characteristics of the PLL treated the m-MoS_2_ TFTs. It is shown that after PLL treatment, the transfer curves shifted left in dark conditions, compared to the bare m-MoS_2_ TFTs. Moreover, the currents of the PLL-treated m-MoS_2_ TFTs under illumination were also higher than those of the bare m-MoS_2_ TFTs, which might be attributed to enhancement of the photoresponse. However, the increased currents of the PLL-treated m-MoS_2_ TFTs under illumination than the bare m-MoS_2_ TFTs might be regarded as only the results of V_th_ shift. Therefore, to clearly confirm the improvement in the photoresponse, photocurrents were extracted in Figure 8d. The larger photocurrents of the PLL-treated m-MoS_2_ TFTs were evidently observed under all wavelength ranges. In addition, the μ_FE_ of the bare m-MoS_2_ TFTs was augmented at 33% under illumination of 365 nm compared to dark conditions, whereas the PLL-treated m-MoS_2_ TFTs had a 92% enhanced μ_FE_ under illumination of 365 nm. Lastly, responsivity and detectivity were examined as quantitative evaluations of photoresponse. Figure 8e presents 2~6 times higher responsivities and 1.3~3.5 times enlarged detectivities of the PLL-treated m-MoS_2_ TFTs, compared to the bare m-MoS_2_ TFTs. These behaviors are well matched with previous results [43,44,45,47]. As a result, Figure 8 validates the improved photoresponse of the m-MoS_2_ TFTs after PLL treatment, which is possibly due to the increased number of tightly bound trions and their lifetime as shown in Figure 7a [46,57].

In addition, the response and recovery time for the m-MoS_2_ and a-Si:H TFTs were extracted at 90% and 10% of maximum photo current. Overall, bare m-MoS_2_ TFTs had a rise (or decay) time in the range from 3.5 (or 6.1) to 7.4 (or 6.9) s, respectively, under wavelengths from 365 to 740 nm, whereas PLL-treated m-MoS_2_ TFTs possessed rise (or decay) time from 2.6 (or 7.9) to 6.1 (or 9.8) sec of rise time, respectively. The shortened response time and prolonged recovery time might be attributed to the enhanced formation of tightly bound trions with PLL treatment in the m-MoS_2_ TFTs [46], leading to a decreased recombination rate of excitons. Reduced PL intensities of the m-MoS_2_ TFTs with PLL treatment supports the decreased recombination rate of excitons as shown in Figure 7a. Therefore, the prolonged lifetime of carriers can induce faster response times of photoexcited carriers and their slower recovery time with PLL treatment. However, response and recovery time of a-Si:H TFTs were extracted within a 10 ms range, which is consistent with other literature [31]. Thus, response and recovery time of the m-MoS_2_ TFTs remained as a problem to be resolved yet. This should be accompanied with an improvement of material, insulator, and passivation, and interface engineering.

Overall, the performance of m-MoS_2_ photodetector itself is not overwhelmed, as compared with the previously reported data in the literature. However, in this study, it is meaningful that the development of generally applicable doping methodology for performance improvement of TMDC photodetectors and its comparative study with conventional products of a-Si:H TFTs in order to understand the figures of merit quantitatively. The present works could provide the intuitive criteria from the perspective of researchers in terms of key aspects that are required for MoS_2_ layers as one of the next-generation semiconductors for the future photodetectors in display systems.

## 4. Conclusions

In this study, a comparative study of the photoresponse of m-MoS_2_ and a-Si:H TFTs was conducted to provide better insight into the photodetector performances of m-MoS_2_ TFTs as photosensors integrated with display systems. With extracted 2~4 orders larger photo responsivity and detectivity of m-MoS_2_ TFTs, it revealed better performance of m-MoS_2_ photodetectors in the various wavelengths and optical power ranges. Furthermore, the photosensitive m-MoS_2_ DLED inverter showed robust advantages with regard to level of device integration per area, chip density, and sensitive modulation properties in variations of wavelength from dark to blue as compared to an a-Si:H DLED inverter. In addition, as a strategy to improve the field effect mobility and photoresponse of the m-MoS_2_ TFTs, molecular doping by PLL treatment was applied to the m-MoS_2_ TFTs. Transfer and output characteristics of the m-MoS_2_ TFTs clearly showed improved photocurrent generation under a wide range of illuminations (740~365 nm), and its chemical and physical properties were analyzed by XPS, Raman spectroscopy, and AFM to confirm the doping effects on m-MoS_2_ TFTs. As a result, along with extracted photo responsivity and detectivity, PLL treatment demonstrated not only the ability to induce n-doping effects but also to improve the photoresponse of the m-MoS_2_ TFTs. This comparative study will provide an intuitive criteria for m-MoS_2_ TFTs to be utilized as future photodetectors in flat panel displays, and it will make them more attractive with their potential for high-performance photodetection enabled by a novel doping technique.

## Data Availability

The data presented in this study are available on request from the corresponding author.
